# Response Characteristics and Experimental Study of Underground Magnetic Resonance Sounding Using a Small-Coil Sensor

**DOI:** 10.3390/s17092127

**Published:** 2017-09-15

**Authors:** Shengwu Qin, Zhongjun Ma, Chuandong Jiang, Jun Lin, Yiguo Xue, Xinlei Shang, Zhiqiang Li

**Affiliations:** 1 College of Construction Engineering, Jilin University, Changchun 130026, China; mzjgeology@gmail.com; 2 College of Institute of Instrument Science and Electrical Engineering, Jilin University, Changchun 130026, China; chuandongjiang@gmail.com (C.J.); lin_jun@jlu.edu.cn (J.L.); shangxl07@mails.jlu.edu.cn (X.S.); 3 Key Laboratory of Geophysics Exploration Equipment, Ministry of Education of China, Changchun 130026, China; 4 Geotechnical and Structural Engineering Research Center, Shandong University, Jinan 250061, China; xieagle@sdu.edu.cn (Y.X.); lzqhlxy@163.com (Z.L.)

**Keywords:** magnetic resonance sounding, small-coil sensor, underground engineering, detection

## Abstract

Due to its unique sensitivity to hydrogen protons, magnetic resonance sounding (MRS) is the only geophysical method that directly detects water and can provide nondestructive information on subsurface aquifer properties. The relationship between the surface MRS signal and the location and characteristics of aquifers using large-coil (typically 50–150 m) sensors has been discussed based on forward modelling and experiments. However, few researchers have studied underground MRS using a small-coil sensor. In this paper, a parametric study and a large-scale physical model test were conducted to shed light on the critical response characteristics of underground MRS using a small-coil sensor. The effects of the size and number of turns of the transmitter coil and receiver coil, the geomagnetic declination, the geomagnetic inclination, and the position, thickness, and water content of a water-bearing structure on the performance of the underground MRS were studied based on numerical simulations. Furthermore, we derived the kernel function and underground MRS signal curves for a water-bearing structure model based on the simulations. Finally, a large-scale physical model test on underground MRS using a small-coil sensor was performed using a physical test system for geological prediction of tunnels at Shandong University. The results show that the inversion results of the physical model test were in good agreement with the physical prototype results. Using both numerical modeling and physical model tests, this study showed that underground MRS using a small-coil sensor can be used to predict water-bearing structures in underground engineering.

## 1. Introduction

Magnetic resonance sounding (MRS) is the only active, non-invasive geophysical technique that provides information on the physical properties of water-bearing structures with an inherent selectivity to free hydrogen, and this technique has become an increasingly popular tool in hydro-geophysics [1,2,3]. Building on the idea of surface MRS proposed in an U.S. patent from the early 1960s [4], the work of Semenov and others in the 1970s, and the robust instruments designed and constructed in the 1980s by Russian scientists [5], the MRS method has been extensively developed over recent decades [6]. This method has been applied mainly, but not exclusively, to assessing the water content and aquifer parameters of primary aquifers.

Many research papers have presented extensive studies on surface MRS and its applications. Among the important contributions on this topic, the following studies have been particularly worthy of mention: Yaramanci et al. [7] obtained the geometry, water content, and hydraulic conductivity of an aquifer in a test at the Nauen/Berlin test site. Legchenko et al. [8] presented the basic principles of the MRS method based on numerical modelling and field examples. Wattanasen et al. [9] succeeded in detecting groundwater using MRS techniques in two areas in southern Sweden. Moreover, Cristina Pérez-Bielsa [10], Boucher [11], Chalikakis [12], and others have made outstanding contributions to the application of MRS in groundwater exploration.

The currently used MRS instruments, including Hydroscope [13], NUMIS [14], SNMR MINI [15], GMR [16], and JLMRS [17], use a circular wire coil with a diameter of 100 m positioned on the ground to excite and receive the MRS signal. When using a square coil configuration, the length of each side of the square is approximately 50–150 m. Trushkin [18] used a coil with a figure-8 shape, which consisted of two connected coils, each with a diameter of 50 m, to eliminate electromagnetic noise. The relationship between the surface nuclear magnetic resonance (NMR) signal and the location and characteristics of aquifers based on large-coil MRS sensors (typically with a coil diameter of 50–150 m) has been discussed based on forward modelling and experiments by Trushkin [18], Weichman [19] Yaramanci [7], Legchenko [20,21], Roy [2], Vouillamoz [22], Lehmann [23], and Hertrich [24]. However, due to the narrow space of underground engineering, it is impossible to use large-coil MRS sensors, and only small-coil MRS sensors can work in a narrow space. Currently, few scientists have addressed the use of small-coil MRS sensors (with a coil diameter of less than 10 m) in underground engineering. The most recent articles have proposed the concept of effective inclination [1], which combines the geomagnetic inclination, declination, and coil angle. The MRS signal is associated with only the effective inclination in space, which simplifies the MRS signal calculations. However, an effective MRS signal cannot be obtained in a mine because of the high noise. Therefore, the authors concluded that the MRS method could not be applied underground. This investigation is the only study on small-coil MRS sensors in the literature.

The main objective of this research is to show how crucial parameters, such as (i) the sizes of the transmitter coil (Tx) and receiver coil (Rx), (ii) the number of turns of Tx and Rx, (iii) the geomagnetic declination and inclination, (iv) the position of the water-bearing structure, (v) the thickness of the water-bearing structure, and (vi) the water content of the water-bearing structure, influence the underground performance of small-coil MRS sensors. In addition, this research explores the response characteristics of underground MRS using a small-coil sensor through a parametric study and a large-scale physical model test. Furthermore, we derive the kernel function and underground MRS response curves of the water-bearing structure model based on the simulations. Finally, a large-scale physical model test is performed, and the results show that the inversion results of the physical model test are in good agreement with the results from the physical prototypes. Thus, underground MRS using a small-coil sensor can be used to predict water-bearing structures in underground engineering.

## 2. Method and Principles

**Figure 1:**
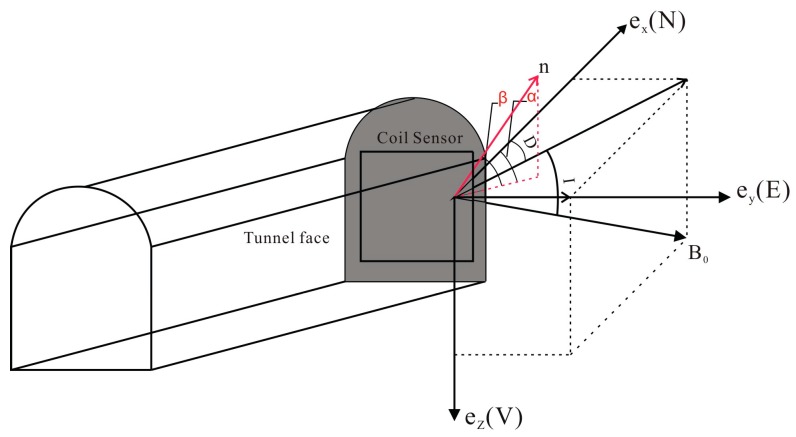
Schematic diagram of underground magnetic resonance sounding (MRS) excitation field and geomagnetic field.

The detailed principles of surface MRS are described in the literature [2,7,8,14,19,20,21,25], and the underground MRS method was developed based on the basic principle of surface MRS [26]. As shown in Figure 1, the coordinate system is defined as follows: ${\vec{e}}_{x}$ is the northern direction, ${\vec{e}}_{y}$ is the eastern direction, and ${\vec{e}}_{z}$ is the vertical direction, where down is positive. The direction of the geomagnetic field ${\vec{B}}_{0}$ is usually represented by the geomagnetic declination D and inclination I.

(1) $${\vec{B}}_{0}=\cos D\cos I{\vec{e}}_{x}+\sin D\cos I{\vec{e}}_{y}+\sin I{\vec{e}}_{z}$$

Only the vertical component of the transmitting field ${\vec{B}}_{T}^{⊥}$ affects the magnitude of the MRS response. When the coil sensor is laid out on the surface horizontally, the vertical component of the transmitting field ${\vec{B}}_{T}^{⊥}$ is expressed as:

(2) $${\vec{B}}_{T}^{⊥}=B_{x}\left[{\vec{e}}_{x}-\left({\vec{e}}_{x}\cdot {\hat{b}}_{0}\right)\right]+B_{y}\left[{\vec{e}}_{y}-\left({\vec{e}}_{y}\cdot {\hat{b}}_{0}\right)\right]+B_{z}\left[{\vec{e}}_{z}-\left({\vec{e}}_{z}\cdot {\hat{b}}_{0}\right)\right]$$

where $B_{x}$, $B_{y}$, and $B_{z}$ are the components of the transmitting field ${\vec{B}}_{T}$ in the *x* axis, *y* axis, and *z* axis directions, respectively. Then, ${\vec{B}}_{T}^{⊥}$ is expressed as:

(3) $${\left({\vec{B}}_{T}^{⊥}\right)}^{2}={\left(-sD\cdot B_{x}+cD\cdot B_{y}\right)}^{2}+{\left(-sIcD\cdot B_{x}-sIsD\cdot B_{y}+sD\cdot B_{z}\right)}^{2}$$

where s and c represent the sin and cos operators, respectively.

When the Tx and Rx are positioned at the tunnel face, this orientation can be characterized by two angles, α and β:

(4) $$\vec{n}=\cos\beta \left(\cos\alpha {\vec{e}}_{x}+\sin\alpha {\vec{e}}_{y}\right)+\sin\beta {\vec{e}}_{z}$$

where α is the deviation angle from the north toward the east, 0<α<2π, and β is the inclination angle from the horizontal plane downward, 0<β<π.

At this point, the vertical component ${\vec{B}}_{T}^{⊥}$ of the transmitting field at an arbitrary coil sensor position is represented as:

(5) $$\begin{array}{l} {\left({\vec{B}}_{T}^{⊥}\right)}^{2}={\left[\begin{array}{l} \left(-sDc\beta c\alpha +cDs\alpha \right)\cdot B_{x}+\\ \left(sDc\beta s\alpha +cDc\alpha \right)\cdot B_{y}+\left(sDs\beta \right)\cdot B_{z} \end{array}\right]}^{2}+[\left(-sIcDc\beta c\alpha +cIs\beta c\alpha -sIsDs\alpha \right)\cdot B_{x}\\ +\left(sIcDc\beta s\alpha -cIs\beta s\alpha -sIsDc\alpha \right)\cdot B_{y}{+\left(sIcDs\beta +cIc\beta \right)\cdot B_{z}]}^{2} \end{array}$$

Underground, the coil sensor is positioned in the vertical direction. Then, the coil is energized by a pulse of alternating current $I(t)=I_{0}\cos(\omega_{0}t)$. The frequency of the current is equal to the Larmor frequency $f_{L}=\gamma B_{0}/2\pi$ of the protons in the geomagnetic field. Here, γ=0.26752 Hz/nT is the gyromagnetic ratio for free hydrogen protons, and $B_{0}$ is the geomagnetic field amplitude. Values for $B_{0}$ vary from 25,000 nT near the equator to 65,000 nT at high latitudes, resulting in Larmor frequencies of 0.9–3.0 kHz. After the pulse is terminated, the spins return to their equilibrium state. During this process, the spins emit an electromagnetic signal that is picked up by the Rx. The expression of the MRS signal is:

(6) $$E_{0}\left(q\right)=\int_{V}K_{3D}\left(q;r\right)n\left(r\right)d^{3}r$$

where $E_{0}$ is the initial amplitude of the MRS signal; $q=I_{0}\cdot \tau$ is the pulse moment, which is the product of the excitation current and excitation time; n is the water content at point r in space; and $K_{3D}$ is the kernel function, which represents the sensitivity of the surface MRS signal at r and is expressed by the following equation [19,27]:

(7) $$\begin{array}{l} K_{3D}\left(q;r\right)=-\omega_{L}M_{0}\sin\left(\gamma_{p}\frac{q}{I_{0}}\left|{\vec{B}}_{T}^{+}\right|\right)\times \frac{2}{I_{0}}\left|{\vec{B}}_{R}^{-}\right|\cdot e^{i\left(\varsigma_{T}+\varsigma_{R}\right)}\\ \times \left[{\hat{b}}_{R}\cdot {\hat{b}}_{T}+i{\hat{b}}_{0}\cdot \left({\hat{b}}_{R}\times {\hat{b}}_{T}\right)\right] \end{array}$$

where $\omega_{L}$ is the Larmor frequency, $M_{0}$ is the net magnetization of the proton, $\gamma_{p}$ is the proton gyromagnetic ratio, ${\vec{B}}_{T}^{+}$ is the counterclockwise component of the transmitting field with vertical component ${\vec{B}}_{T}^{⊥}$, ${\vec{B}}_{R}^{-}$ is the clockwise component of the receiving field with vertical component ${\vec{B}}_{R}^{⊥}$, and $\varsigma_{T}$ and $\varsigma_{R}$ are the phase components of the elliptical polarization field for transmitting and receiving, respectively. The unit vectors ${\hat{b}}_{T}$, ${\hat{b}}_{R}$, and ${\hat{b}}_{0}$ are the direction vectors of the transmitting field, the receiving field and the geomagnetic field, respectively.

When the coil sensor layout is vertical, the normal direction is the x axis. In this coordinate system, the tunnel axis is the x axis, the excavation direction is the positive x direction, and the location of the tunnel face is 0. The MRS kernel function $K_{3D}\left(q,r\right)$ should be integrated over the range of (−∞,∞) on the y axis and z axis, separately, and then integrated over the range of (−∞,∞) on the x axis to calculate the MRS signal:

(8) $$K_{1D}\left(q,x\right)=\int_{-\infty }^{\infty }\int_{-\infty }^{\infty }K_{3D}\left(q,x,y,z\right)dydz$$

(9) $$E_{0}\left(q\right)=\int_{-\infty }^{\infty }K_{1D}\left(q,x\right)n\left(x\right)dx$$

## 3. Numerical Analysis of Underground MRS Using a Small-Coil Sensor

The factors that influence the MRS signal include the electrical conductivity of the rock; the geomagnetic field; the geomagnetic inclination; the position; thickness and water content of water-bearing structures; and the electromagnetic noise, all of which are naturally occurring properties that have been discussed in many studies [28,29]. In addition, artificial technical factors, such as the shape, size, and number of turns of the detection coil; the maximum value and number of pulse moments; the detection sensitivity; and the inversion method also affect the MRS signal. As the amplitude of the MRS signal directly determines the detection distance, these artificial technical factors can be varied to achieve the optimal design for the coil sensor and instrument system. In addition, the MRS method can be adapted to the specific environments of mines and tunnels to achieve advanced detection of water.

### 3.1. Calculation of the Kernel Function

A numerical study has been conducted to explore the effects of the size and number of turns of Tx and Rx, the geomagnetic declination and the inclination on underground MRS performance. We assume that the resistivity of the surrounding rock is 500 Ω⋅m, that the Larmor frequency is 2300 Hz, and that the coil sensor is positioned vertically in the north-south direction (α=0°，β=0°).

**Figure 2:**
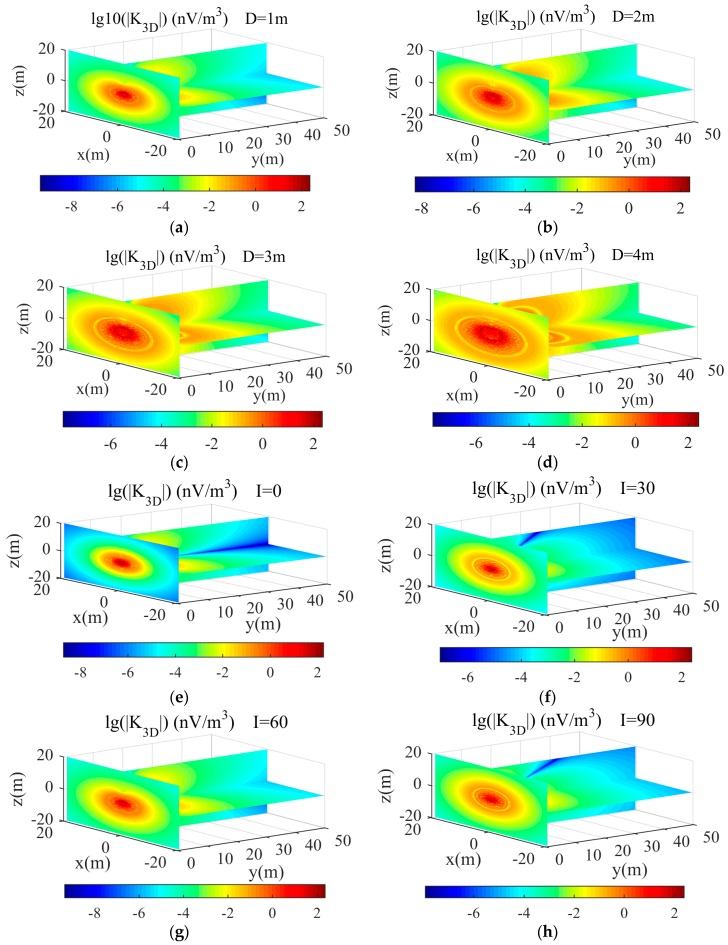

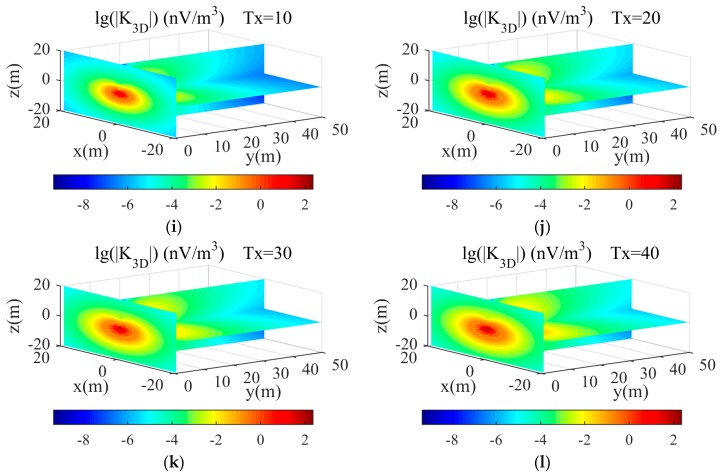
Three-dimensional slices of the kernel model results for underground MRS using a small-coil sensor. The Larmor frequency is 2300 Hz, the resistivity of the surrounding rock is 500 Ω⋅m, and the coil is positioned vertically in the north-south direction (α=0°，β=0°). (**a**–**d**) Kernel model results for coil diameters of 1 m, 2 m, 3 m, and 4 m, respectively. 40 turns are assumed for Tx, and 128 turns are assumed for Rx; the geomagnetic inclination is 60°, and the geomagnetic declination is 0°. (**e**–**h**) Kernel model results using a 1-m diameter coil for geomagnetic inclinations of 0, 30, 60, and 90°, respectively. 40 turns are assumed for Tx, and 128 turns are assumed for Rx; the geomagnetic declination is 0°. (**i**–**l**) Kernel model results using a 1-m diameter coil for 10, 20, 30, and 40 coil turns for Tx, and 128 turns are assumed for Rx, respectively. The geomagnetic inclination is 60°, and the geomagnetic declination is 0°.

Figure 2 shows the three-dimensional slices of the kernel model results varying three different parameters. The three-dimensional coordinates are vertical distance, horizontal distance, and front depth, in meters. Figure 2a–d clearly shows the forward kernel model results for coil diameters of 1 m, 2 m, 3 m and 4 m, respectively. The detection depth increases with increasing coil diameter, and the asymmetry of the kernel model results decreases with decreasing coil diameter. In addition, the forward kernel models change irregularly because of the inhomogeneous magnetic field.

Figure 2e–h illustrates the forward kernel models for geomagnetic inclination angles of 0°, 30°, 60° and 90°, respectively. The results show that the asymmetry of the kernel remains constant as the geomagnetic inclination changes, indicating the important effect of the kernel on these parameters in the modeling process.

Figure 2i–l shows that the influence of varying the number of coil turns from 10 to 40. The results clearly demonstrate that with an increasing number of coil turns, the asymmetry of the kernel forward models increases. When the number of coil turns is larger than 40, the detection depth is more than 10 m, i.e., the instrument can receive a valid MRS signal at 10 m. Comparing Figure 2a–d and Figure 2i–l, the forward modelling results of the two graphs show basically the same trend. Thus, increasing the size of the detection coil sensor and increasing the number of turns of the coil sensor improves the effective area of the detection coil.

### 3.2. Underground MRS Response

To perform an exploratory analysis on the response regularity and to understand the effects of the position, thickness and water content of water-bearing structures on underground MRS using a small-coil sensor, the following simulations were performed to simulate the MRS signal using Equations (5), (8) and (9). We modeled three groups of water-bearing geological structures ahead of the tunnel face with different positions, thicknesses and water contents based on the advanced detection theory of the underground MRS method, and we calculated the underground MRS signal curves. We assumed that the earth resistivity is 500 Ω⋅m, the Larmor frequency is 2300 Hz, a 1-m diameter coil is positioned vertically in the north-south direction (α=0°，β=0°), Tx has 40 turns, Rx has 128 turns, the geomagnetic inclination is 60° and the geomagnetic declination is 0°. For the first model, a water-bearing structure 2 m thick is located 5 m behind the coil with varying water content of 25%, 50%, 75%, and 100%. For the second model, a water-bearing structure 2 m thick is located 10 m behind the coil with varying water content of 25%, 50%, 75%, and 100%. For the third model, the water-bearing structure 1 m thick is located 10 m behind the coil with varying water content of 25%, 50%, 75%, and 100%.

**Figure 3:**
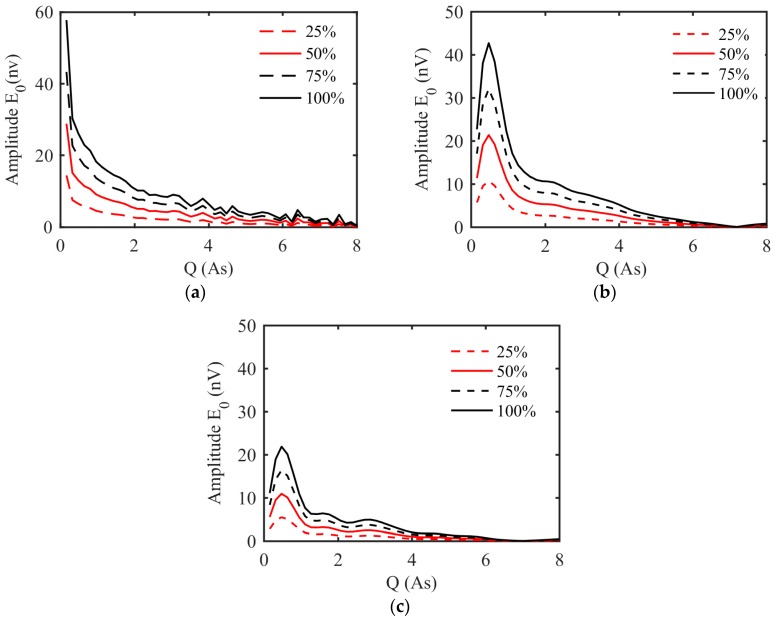
The underground MRS response $E_{0}-q$ curves from the forward model assuming a 1-m diameter coil, 40 turns for Tx and 128 turns for Rx. (**a**) a 2-m-thick water-bearing structure located 5 m ahead of the tunnel face with varying water content of 25%, 50%, 75%, and 100%; (**b**) a 2-m-thick water-bearing structure located 10 m ahead of the tunnel face with varying water content of 25%, 50%, 75%, and 100%; (**c**) a 1-m-thick water-bearing structure located 10 m ahead of the tunnel face with varying water content of 25%, 50%, 75%, and 100%.

The response regularity of the underground MRS is illustrated in Figure 3. As shown in Figure 3a,b, when the position of the water-bearing structure remains constant and the water content increases from 25% to 100%, the amplitude of the $E_{0}-q$ curves for a 2-m-thick water body located 5 m ahead of the tunnel face increases from 14.43 nv to 57.74 nv, and that for a 2-m-thick water-bearing structure 10 m ahead of the tunnel face increases from 10.67 nv to 42.69 nv. In addition, the abscissa values corresponding to the peak value of the $E_{0}-q$ curve remains unchanged at 0.16 As and 0.48 As, respectively. Figure 3c shows the $E_{0}-q$ curves that were generated using a model with a 1-m-thick water-bearing structure located 10 m ahead of the tunnel face with varying water content of 25%, 50%, 75%, and 100%. Figure 3a,b clearly show that the Q value at the $E_{0}$ peak increases from 0.16 As to 0.48 as the depth of the water-bearing structure increases from 5 m to 10 m ahead of the tunnel face. Comparing Figure 3b,c, the Q value at the $E_{0}$ peak remains unchanged, and the peak of the $E_{0}-q$ curve decreases with decreasing thickness of the water-bearing structure.

To explore the effects of the number of turns and the size of Tx and Rx, the geomagnetic declination and the inclination on underground MRS using a small-coil sensor, we ran the following simulations to simulate the underground MRS signal using Equations (5), (8) and (9). We assumed a 2-m-thick water-bearing structure located 10 m ahead of the tunnel face with a water content of 100% and an earth resistivity of 500 Ω⋅m.

**Figure 4:**
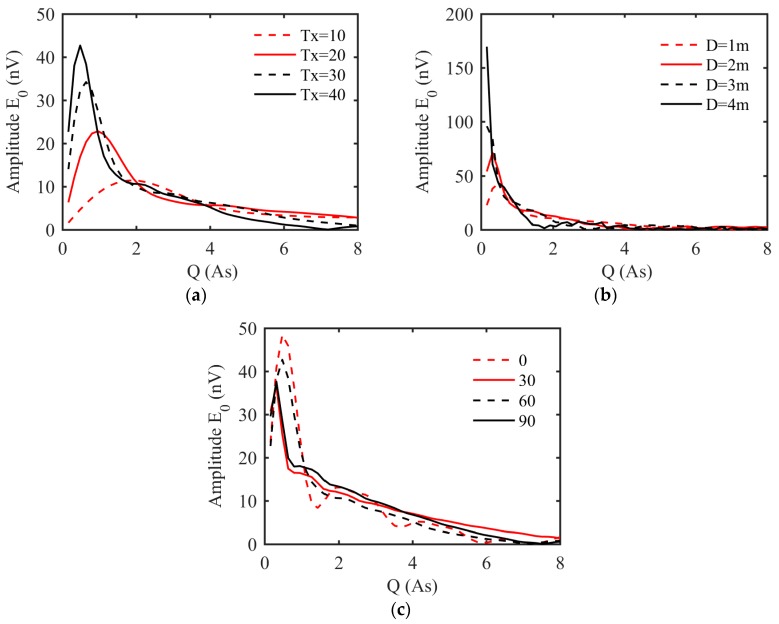
The underground MRS response $E_{0}-q$ curves from the forward model for a 2-m-thick water-bearing structure located 10 m ahead of the tunnel face with a water content of 100% (**a**) results assuming a 1-m-diameter coil; 128 turns for Rx; 10, 20, 30, or 40 turns for Tx; geomagnetic inclination of 60°; and geomagnetic declination of 0°; (**b**) results assuming 40 turns for Tx; 128 turns for Rx; coil diameter of 1, 2, 3, or 4 m; geomagnetic inclination of 60°; and geomagnetic declination of 0°; (**c**) results assuming a 1-m-diameter coil, 40 turns for Tx, 128 turns for Rx, and geomagnetic inclination of 0, 60, or 90°.

Figure 4a shows the $E_{0}-q$ curves that were generated using a model of a 2-m-thick water-bearing structure located 10 m ahead of the tunnel face with a water content of 100% and 10, 20, 30, or 40 turns for Tx. As shown in the figure, gradually increasing the number of turns of Tx increases the maximum $E_{0}$ from 11.3 nvto 42.69 nv and moves the wave crest of the $E_{0}-q$ curve to the left. Thus, when the radius of the coil is fixed and the number of turns increases, the excitation pulse moment needed to detect an aquifer of a certain depth can be decreased accordingly.

Figure 4b shows the $E_{0}-q$ curves that were generated using a model of a 2-m-thick water-bearing structure located 10 m ahead of the tunnel face with a water content of 100% and a coil diameter of 1, 2, 3, or 4 m. As shown in the figure, as the coil diameter gradually increases, the maximum initial amplitude of the signal becomes larger and moves to the left in the direction in which the value decreases. Thus, as the diameter of the coil sensor increases, the excitation pulse needed to detect a water-bearing structure at a certain depth can be decreased accordingly.

Figure 4c shows the $E_{0}-q$ curves that were generated using a model for a 2-m-thick water-bearing structure located 10 m ahead of the tunnel face with a water content of 100% and a geomagnetic inclination of 0°, 60°, or 90°. The curves clearly change with varying inclination, and the same results can be found in previous studies [1,24]. The maximum $E_{0}$ decreases with increasing inclination.

**Figure 5:**
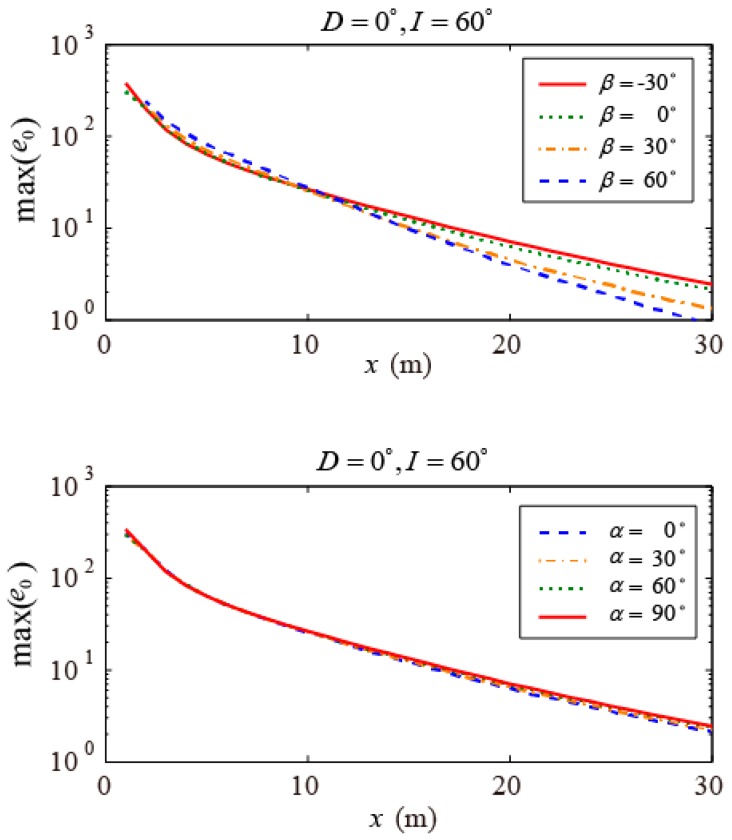
Maximum values of MRS signals with varying coil directions for a 1-m-thick aquifer with a water content of 100% located at varying distances

Next, simulations were run to explore the effect of the coil direction (α and β) on underground MRS using a small-coil sensor. This model assumed a square coil with a side length of 2 m and 100 turns, a geomagnetic inclination of $I=60^{\circ }$ and a declination of $D=0^{\circ }$, and a variation in the normal direction of the coil ($\beta =-30^{\circ }\sim 60^{\circ }$ and $\alpha =0^{\circ }\sim 90^{\circ }$). The maximum amplitudes of the MRS signal generated by a 1-m-thick aquifer with a water content of 100% located at a distance of 1 m to 30 m ahead of the tunnel face are calculated and presented in Figure 5. As β increases from $-30^{\circ }$ to $60^{\circ }$, the maximum amplitude of the MRS signal from the aquifer within 10 m increases gradually. This result means that when the normal direction of the coil is parallel to the direction of the geomagnetic field, the MRS signal reaches its maximum value; in contrast, when they are perpendicular to each other, the MRS signal reaches its minimum value. However, for an aquifer located at a distance of greater than 10 m, the opposite results are found, i.e., the value of the MRS signal reaches its maximum when the two directions are perpendicular and reaches its minimum when they are parallel. Moreover, as α changes from $0^{\circ }$ to $90^{\circ }$, the value of the MRS signal remains nearly constant, except for an aquifer located far away, where it is slightly larger when the geomagnetic field and the coil are perpendicular than when they are parallel. Therefore, the amplitude of the MRS signal can be improved by changing the coil direction. For a close aquifer, the coil should be positioned parallel to the geomagnetic field, while for a distant aquifer, the coil should be perpendicular.

## 4. Physical Model Test

As a direct and quantitative advanced geophysical prediction method, the advanced prediction of underground MRS is an important reference for quantitatively predicting waterborne geological bodies in tunnels. The purpose of this experiment is to explore the relationship between the MRS signal and the water content of the water-bearing structure ahead of the tunnel face and to explore the feasibility of using a small-coil sensor in the advanced prediction of underground MRS.

### 4.1. Forward Model

**Figure 6:**
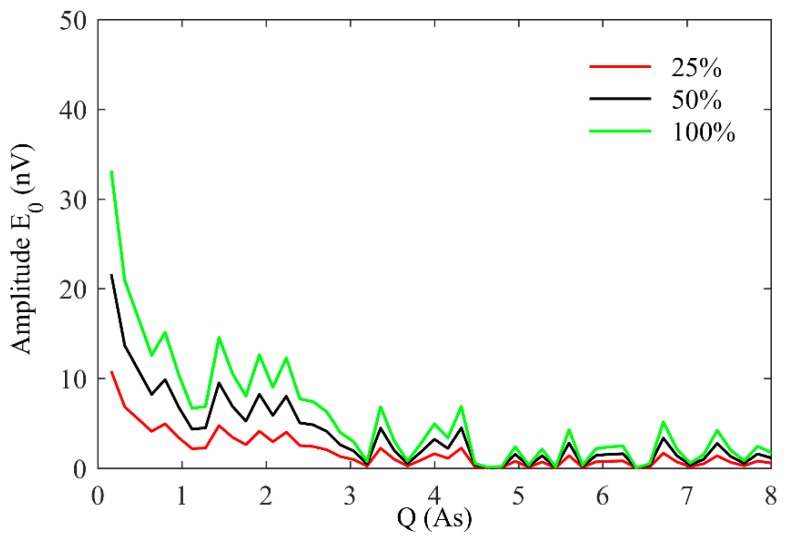
Underground MRS response curve of $E_{0}-q$ with varying water content of 25%, 50% and 100%.

Prior to the physical model test, to explore the feasibility of underground MRS using a small-coil sensor, three groups of water-bearing geological models were established. The parameters of the hydrogeological models are shown in Table 1, and the response $E_{0}-q$ curves were calculated and are shown in Figure 6. We assume the following parameters: earth resistivity of 500 Ω⋅m, Larmor frequency of 2300 Hz, a square coil with a side length of 1 m placed vertically in the north-south direction (α=0°，β=0°), 40 turns for Tx, 128 turns Rx, geomagnetic inclination of 60° and geomagnetic declination of 0°. As shown in Figure 6, the maximum $E_{0}$ increases from 10.81 nv to 33.12 nv as the water content increases from 25% to 100%.

### 4.2. Physical Model Test Analysis

**Figure 7:**
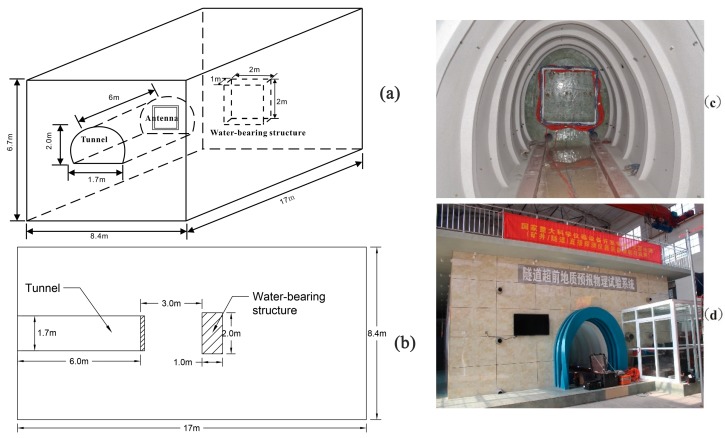
Diagram of the model tunnel and water-bearing structure. (**a**) 3-D diagram; (**b**) longitudinal profile of the test model; (**c**) small-coil sensor; (**d**) physical test system for geological prediction of tunnels.

To simulate the underground MRS using a small-coil sensor in an actual project, an experiment was conducted using a large-scale experimental device. The tested system is based on the JLMRS instrument [17] and a small-coil sensor. A schematic diagram of the large physical model tests is shown in Figure 7. The large-scale integrated geophysical advanced detection model test device is 17 m long, 8.4 m wide, and 6.7 m high. The tunnel model is located in the middle of the large-scale model and is 2.0 m high, 1.7 m wide and 6 m long. To reduce the influence of the reinforcement and other factors on the test results, the tunnel cavity model is made of glass fiber-reinforced plastic materials, and clay is used as the filling material to represent the surrounding rock. The water-bearing geological structure device is placed 3 m ahead of the main tunnel model and has dimensions of 1.0 m × 2.0 m × 2.0 m (thickness × width × height), which is consistent with the cavities that often appear in tunnel construction. We used a square detection coil (1 m × 1 m with 40 turns for Tx and 128 turns for Rx) to perform detections within the tunnel model. The water-bearing structure was gradually filled with water at water levels of 1 m, 1.5 m, and 2 m.

**Figure 8:**
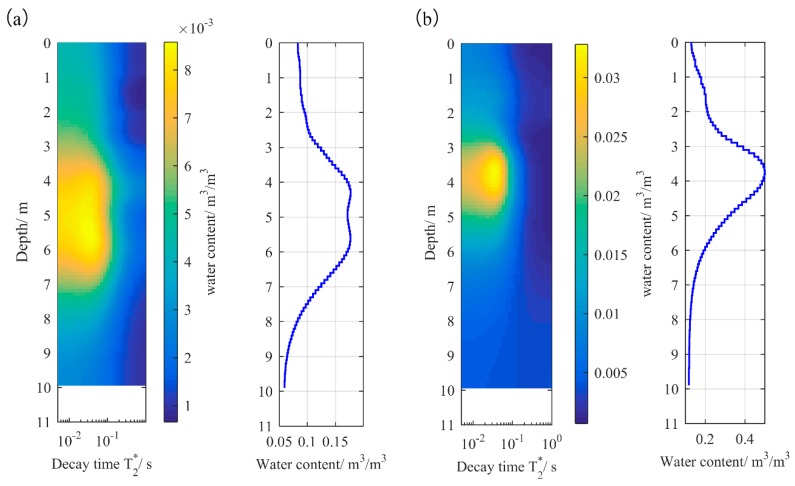

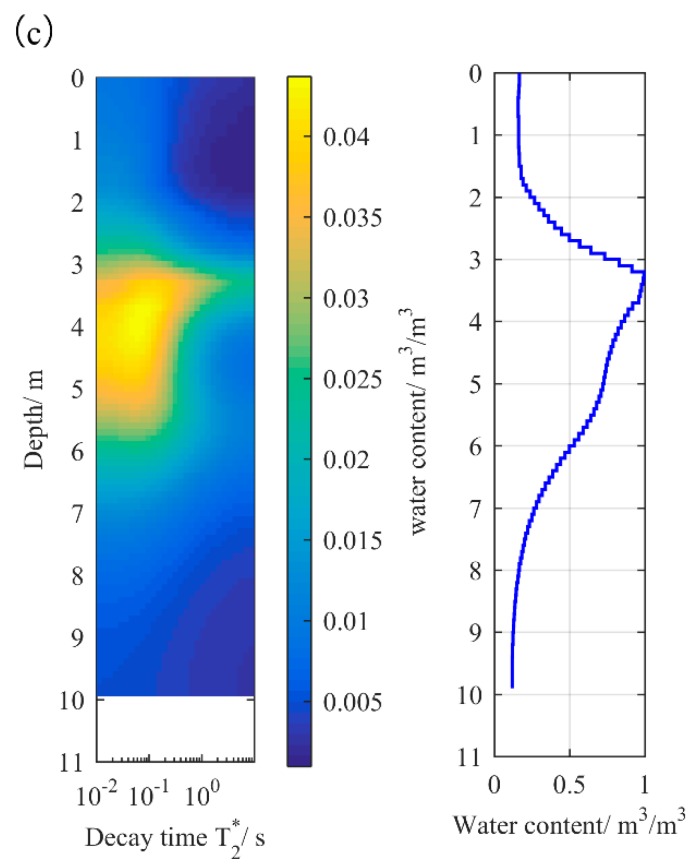
The inversion results of underground MRS. (**a**) inversion results for a water level of 1.0 m; (**b**) inversion results for a water level of 1.5 m; (**c**) inversion results for a water level of 2.0 m.

As shown in Figure 8, the water curves are obtained using the QT inversion method [30,31]. The results shown in Figure 8c are the underground MRS inversion results for water levels of 1 m, 1.5 m, and 2 m, respectively. Thus, the relationship between the underground MRS signal and the water level is obtained.

(1) The small-coil sensor used in underground MRS can receive valid MRS signals in the prediction experiment.
(2) As the water level increases from 1 m to 2 m, the peak value of the water content curve increases from 0.18 to 1. Thus, the underground MRS technique can effectively quantify the size of the water-bearing structure.
(3) The inversion results can accurately locate the aquifer, and the location and water content of the aquifer are consistent with the physical model, which confirms the validity of the prediction.

## 5. Conclusions

For this study, experimental and numerical approaches are used to accurately analyze an underground MRS using a small-coil sensor. The factors that influence the MRS signal include the size and number of turns of Tx and Rx; the geomagnetic declination; the geomagnetic inclination; and the position, thickness and water content of the water-bearing structure, all of which have been previously studied. The response characteristics of underground MRS using a small-coil sensor are obtained based on a parametric numerical study. The forward model results show that with increasing model depth, the amplitude decreases, and larger pulse moments are needed. With increasing water content and thickness of the water-bearing structure, the initial signal amplitude $E_{0}$ increases. We also conclude that the detection depth and the initial signal amplitude $E_{0}$ increase as the size and number of turns of the detection coil sensor increase, and both of these factors increase the effective detection area.

A large-scale physical model test using a small-coil sensor is performed using the test system for geological prediction of tunnels. The small-coil sensor successfully detected a valid MRS signal, and the inversion results of the physical model test are in good agreement with the physical prototype results, which verifies the feasibility and effectiveness of the predictions of underground MRS using a small-coil sensor. Using both numerical modeling and physical model tests, this study shows that underground MRS using a small-coil sensor can be used to predict water-bearing structures in underground engineering.

## Figures and Tables

**Table 1:** Parameters of the theoretical models

Number	Aquifer Location (m)	Aquifer Thickness (m)	Water Content (%)
a	3	1	25
b	3	1	50
c	3	1	100
